# Improving nitrogen contribution in maize post-tasseling using optimum management under mulch drip irrigation in the semiarid region of Northeast China

**DOI:** 10.3389/fpls.2022.1095314

**Published:** 2022-12-09

**Authors:** Yunpeng Hou, Xinpeng Xu, Lili Kong, Lei Zhang, Yitao Zhang, Zhiquan Liu

**Affiliations:** ^1^ Institute of Agricultural Resources and Environment Research, Jilin Academy of Agricultural Sciences, Changchun, China; ^2^ Key Laboratory of Plant Nutrition and Fertilizer, Ministry of Agriculture and Rural Affairs/Institute of Agricultural Resources and Regional Planning, Chinese Academy of Agricultural Sciences, Beijing, China; ^3^ Institute of Geographic Sciences and Natural Resources Research, Chinese Academy of Sciences, Chinese Academy of Agricultural Sciences, Beijing, China

**Keywords:** Maize, film-mulched drip irrigation, semiarid Northeast China, nitrogen use efficiency, nitrogen loss

## Abstract

Film-mulched drip irrigation has become an important strategy in maize cultivation in the semiarid region of Northeast China. Most farmers concentrate nitrogen (N) fertilizer use early in maize growth, which leads to low N use efficiency and large N losses. Therefore, a three-year (2018 to 2020) field experiment was conducted to determine the optimal N management strategy for maize under film mulch with drip irrigation in the semiarid region of Northeast China. The experiment included five treatments with the total amount of N fertilizer (210 kg N ha^−1^) applied in different proportions at sowing, sixth-leaf (V6), twelfth-leaf (V12), tasseling (VT), and blister (R2) stages of maize growth: N1, 100-0-0-0-0; N2, 50-50-0-0-0; N3, 30-50-20-0-0; N4, 20-30-30-20-0; and N5, 10-20-30-20-20. The control (CK) did not receive N fertilizer. Maize yield, N uptake and use, changes in soil inorganic N content, and N balance were investigated. Compared with the single basal application (N1), split-N applications (N2, N3, N4, N5) increased maize yield from 13.8% to 24.5% by increasing kernel number per ear and 1000-kernel weight and also improved N accumulation from VT to physiological maturity (PM) stages and its contribution to grain N uptake. In addition, compared with N1, split-N applications also decreased N losses by increasing inorganic N contents in the 0–40 cm soil layer and by decreasing N leaching in the 60–200 cm soil layer. Regression analysis demonstrated that N accumulation after the VT stage was positively related with maize yield. Among treatments, N4 had the highest yield, N recovery efficiency, agronomic efficiency, and partial factor productivity, with respective increases of 24.5%, 14.7 percentage point, 11.4 kg kg^−1^, and 11.4 kg kg^−1^ compared with those in N1. As a result, N losses were also reduced by 33.7% in N4 compared with those in N1. In conclusion, the split-N management strategy with four N applications under film-mulched drip irrigation has great potential to improve maize yield, increase N use efficiency, and reduce N loss in the semiarid region of Northeast China.

## 1 Introduction

Nitrogen (N) fertilization supplies the most significant nutrient to increase crop yields and ensure food security (Erisman et al., 2008). However, environmental problems caused by excessive N fertilizer input must also be addressed, which because of low N use efficiency (Dobermann and Cassman, 2005; Wang et al., 2011; Ma et al., 2012), include pollution of groundwater and surface water and emissions of greenhouse gases due to N leaching, runoff, and volatilization (Gheysari et al., 2009; Schroder et al., 2011; Yan et al., 2013; Jia et al., 2014; Pan et al., 2016; Pareja-Sánchez et al., 2020).

Effective N management with the “4R” strategy (right source, right rate, right time, right place) has been advocated to ensure synchronization between soil N supply and crop N requirements and thereby maximize crop yield and N use efficiency and minimize negative environmental effects (Malhi et al., 2001; Cui et al., 2010). Split-N fertilizer management increases yield and N use efficiency by improving crop N uptake (Randall et al., 2003; Walsh et al., 2012) and reducing N losses (Abbasi et al., 2013). However, split fertilizer application also increases machinery and labor costs (Torbert et al., 2001; Otteson et al., 2007; Patil et al., 2010), and fertilizer application is difficult in middle and late stages of crop growth. In fact, it is a great challenge to apply multiple topdressings according to crop N demand during production, particularly with the continued decrease in the agricultural labor force (Grant et al., 2012; Zheng et al., 2017).

The western region of Northeast China is a semiarid agricultural region with 3.2 × 10^7^ ha of arable land, and it is one of the important maize growing regions in China (China Agriculture Statistical Report, 2021). However, maize yields in the region are lower than those in areas with sufficient moisture because of low temperatures and drought in early spring (Campos et al., 2004; Li et al., 2019). In recent years, mulch drip irrigation, which combines mulch planting and drip irrigation, has been implemented as a water-saving irrigation technology. Because it greatly improves soil hydrothermal conditions, promotes crop growth and development, and increases water and fertilizer use efficiency (Kong et al., 2020), mulch drip irrigation was introduced and has become one of the major agricultural extension technologies in semiarid western Northeast China (Zhou et al., 2009; Sharma et al., 2011; Qin et al., 2016). Although drip irrigation can accelerate growth and promote nutrient transformation (Liu et al., 2014), most farmers continue to follow the traditional fertilization pattern in which most N fertilizer is applied in a basal application rather than according to crop N demand. This traditional practice reduces effective soil N in late growth stages and ultimately reduces maize yield and N use efficiency (Liu et al., 2010; Liu et al., 2015).

In the semiarid region of Northeast China, drip irrigation is usually required four to five times to meet maize water requirements during different growing periods. Thus, it is important to identify N and fertilizer transport schemes applicable to mulching combined with drip irrigation. In addition, to optimize management measures for maize in semiarid and arid areas, it is also important to further explore the coupling of mulching, drip irrigation, and N fertilizer. However, research on N fertilizer application strategies under mulch drip irrigation in this region is lacking. Therefore, it was hypothesized that phased application of N fertilizer under drip irrigation would significantly increase maize yield and N fertilizer use efficiency and reduce N fertilizer losses.

To test the hypothesis, a three-year field experiment was conducted with the total amount of N fertilizer applied in different proportions at different stages of maize growth under film-mulched drip irrigation. The objectives were to (1) determine the effects of split-N applications on maize yield and N use efficiency, (2) analyze changes in soil inorganic N and determine apparent N balance and N losses, and (3) recommend the optimal N fertilizer application strategy for film-mulched drip-irrigated maize in semiarid areas of northeastern China.

## 2 Materials and methods

### 2.1 Experimental site

**Figure 1:**
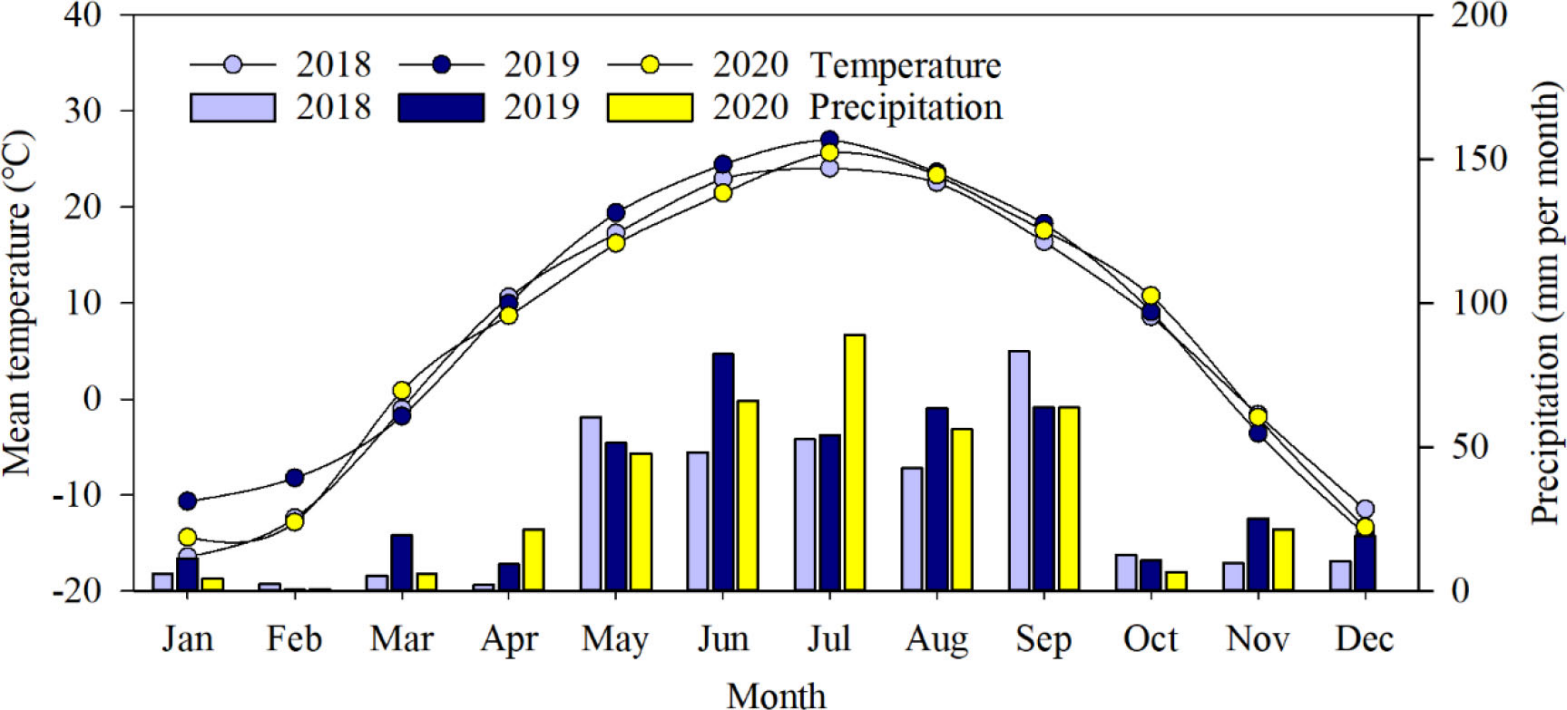
Average monthly temperature (°C) and precipitation (mm) from 2018 to 2020 in an experimental maize field in Northeast China.

The field experiment was conducted from 2018 to 2020 at the experimental station of Jilin Academy of Agricultural Sciences, Qian’an County, Jilin Province, Northeast China (44°39′21″N, 123°35′10″E). The station is in the northwestern part of Jilin Province, which has a cool temperate climate with a single cropping season. Average annual precipitation is 400 mm, and average annual evaporation is greater than 1,500 mm (**Figure 1**). Initial soil chemical properties at 0–20 cm were the following: organic matter 17.4 g kg^−1^; total N 1.2 g kg^−1^; Olsen-phosphorus (P) 14.98 mg kg^−1^; ammonium acetate-potassium (K); and pH 8.06. Before planting, the soil nitrate-N (NO_3_^−^–N) and ammonium-N (NH_4_^+^–N) concentrations were 16.91 and 3.13 mg kg^−1^ at 0–20 cm; 15.28 and 3.18 mg kg^−1^ at 20–40 cm; 11.28 and 2.92 mg kg^−1^ at 40–60 cm; 9.32 and 3.11 mg kg^−1^ at 60–80 cm; 8.39 and 2.66 mg kg^−1^ at 80–100 cm; 8.93 and 2.96 mg kg^−1^ at 100–120 cm; 7.28 and 3.24 mg kg^−1^ at 120–140 cm; 7.61 and 3.13 mg kg^−1^ at 140–160 cm; 6.35 and 3.17 mg kg^−1^ at 160–180 cm; and 6.27 and 2.73 mg kg^−1^ at 180–200 cm, respectively.

### 2.2 Experimental design and field management

The N fertilizer rate in drip-irrigated maize was 210 kg N ha^−1^, which was determined in a previous study in the region (Hou et al., 2018). The experiment included five treatments with the total amount of N fertilizer (210 kg N ha^−1^) applied in different proportions at sowing, sixth-leaf (V6), twelfth-leaf (V12), tasseling (VT), and blister (R2) stages of maize growth: N1, 100-0-0-0-0; N2, 50-50-0-0-0; N3, 30-50-20-0-0; N4, 20-30-30-20-0; and N5, 10-20-30-20-20 (**Table 1**). The control (CK) did not receive N fertilizer. Each treatment was replicated three times in a completely randomized design. The same amounts of P (80 kg P_2_O_5_ ha^−1^) and K (100 K_2_O ha^−1^) fertilizers were applied in each treatment as basal fertilizer before sowing. Fertilizers were urea (N 46%), calcium superphosphate (P_2_O_5_ 46%), and potassium chloride (K_2_O 60%).

**Table 1:** Proportion of total nitrogen (N) fertilizer and growth stage in which fertilizer was applied in different N treatments in film-mulched drip-irrigated maize in Northeast China.

Treatment	N rate (kg ha^−1^)	Proportion (%) applied per growth stage				
		Sowing	V6	V12	VT	R2
CK	0	0	0	0	0	0
N1	210	100	0	0	0	0
N2	210	50	50	0	0	0
N3	210	30	50	20	0	0
N4	210	20	30	30	20	0
N5	210	10	20	30	20	20

The total amount of N fertilizer applied in different proportions at sowing, sixth-leaf (V6), twelfth-leaf (V12), tasseling (VT), and blister (R2) stages of maize growth: N1, 100-0-0-0-0; N2, 50-50-0-0-0; N3, 30-50-20-0-0; N4, 20-30-30-20-0; and N5, 10-20-30-20-20. CK: no N fertilizer.

Maize variety Huanong101 was planted at a density of 70,000 plants ha^−1^. The cultivation mode was large ridge with double rows under film mulching. Row spacing on the ridge was 40 cm, and row spacing between ridges was 90 cm. Treatment plots were 60 m^2^ (7.5 × 11.53 m, 8 rows per plot). Maize was planted on May 5, May 8, and May 6 and was harvested on September 30, October 2, and October 5 in 2018, 2019, and 2020, respectively. Before mulching, herbicides were sprayed on the soil surface to control weeds. Mulching was synchronized with laying the drip irrigation belt. The plastic film was a polyethylene agricultural transparent film with a width of 120 cm and a thickness of 0.08 mm. Drip irrigation belts used inner inlaid pieces, and spacing of drippers was 30 cm. Drip irrigation belts were placed in the middle of ridges, and each drip irrigation belt watered two rows of maize. Based on a previous study (Geng et al., 2021), the drip irrigation quota for spring maize in western Northeast China was 1,600 m^3^ ha^−1^ per year, with six drip irrigations, including 100 m^3^ ha^−1^ for pre-sowing, 75 m^3^ ha^−1^ for VE (emergence), 225 m^3^ ha^−1^ for V6, 375 m^3^ ha^−1^ for V12, 450 m^3^ ha^−1^ for VT, and 375 m^3^ ha^−1^ for R2 stages. A separate water meter was used to control the irrigation amount in each treatment. Each plot was equipped with a separate 18-L differential pressure fertilizer tank. Duration of fertilizer application was 120 min, with irrigation with water without fertilizer 30 min before and after fertilization. Other field measures were optimized and managed according to local agricultural technicians.

### 2.3 Sampling and analysis

Maize samples were collected from three-leaf (V3), V6, V12, VT, R2, and physiological maturity (PM) stages. In each plot, five plants were collected and divided into straw and grain parts in the 2^nd^ and 7^th^ rows. All samples were fixed at 105°C for 30 min and then dried at 70°C to constant weight. Plant biomass was weighed for each treatment. Plant materials were crushed, and N contents of straw and grain were determined following digestion with H_2_SO_4_–H_2_O_2_ according to a Kjeldahl method.

At harvest, grain yield was measured in 30 m^2^ (between the 3^rd^ and 6^th^ rows) in each plot and ultimately converted to 14% standard water content. In addition, 30 representative plants were selected to measure kernel number per ear and 1000-kernel weight.

Soil samples at 20 cm intervals from 0 to 200 cm were collected before planting in 2018 and after harvesting in 2020. Soil was randomly sampled at eight points, and samples were mixed into one composite sample per plot. To determine water content, soils were oven-dried at 105°C for 24 h. To determine NO_3_^−^-N and NH_4_^+^-N contents, soils were sieved through a 5-mm screen and then extracted using 1 mol L^−1^ KCl solution (soil:liquid ratio 1:5) with shaking. Extracts were analyzed for NO_3_^−^-N and NH_4_^+^-N on a flow injection analyzer (Model Auto Analyzer 3-AA3-HR, SEAL Analytical, Germany). The soil bulk density was determined using the cutting ring method. For each plot, five points were evaluated in the soil profile within 100 cm and cutting-ring soil samples were collected at 20 cm intervals throughout the soil layer. The soil was dried at 105°C for 24 h.

### 2.4 Sampling and analysis

Contribution of accumulated N after the VT stage to grain N accumulation under different split-N treatments was assessed as follows (Chen et al., 2016):


(1) $$\begin{array}{c} NaccumulationafterVTstage\;\left(kgha^{-1}\right)\\ \;=\;plantNaccumulationatPMstageplantNaccumulationatVTstage \end{array}$$

(2) $$\begin{array}{c} ContributionofaccumulatedNafterVTtograinN\;\left(\%\right)\\ \;=\;NaccumulationafterVTstage/plantNaccumulationatPMstage\\ \;\times 100 \end{array}$$

Accumulated recovery efficiency (REN), agronomic efficiency (AEN), and partial factor productivity (PFPN) under different N treatments were calculated as follow:


(3) $${\text{REN}}_{i}=\frac{\sum_{i=1}^{n}\left(U_{Fi}-U_{CKi}\right)}{\sum_{i=1}^{n}F_{Ni}}\times 100\%$$

(4) $${\text{AEN}}_{i}=\frac{\sum_{i=1}^{n}\left(Y_{Fi}-Y_{CKi}\right)}{\sum_{i=1}^{n}F_{Ni}}$$

(5) $${\text{PFPN}}_{i}=\frac{\sum_{i=1}^{n}Y_{Fi}}{\sum_{i=1}^{n}F_{Ni}}$$

where *i* is the season (*i* = 1, 2, …); *U_F_* and *U_CK_* are N uptake in aboveground dry matter with and without N fertilizer application (kg ha^−1^), respectively; *Y_F_* and *Y_CK_* are maize yield with and without N fertilizer application (kg ha^−1^), respectively; and *F_N_* is the N fertilizer application amount (kg ha^−1^).

To evaluate environmental effects of different N application modes, the apparent N balance between N input and output was estimated in the soil–plant systems. Contents of NO_3_^−^-N and NH_4_^+^-N in the top 100 cm of soil were used in N budget calculations. Apparent N mineralization and apparent N loss were determined according to the following formula:


(6) $$N_{min}=\frac{T\times BD\times N_{inorganic}}{10}$$

where T is the thickness of the soil layer (cm); BD is the soil bulk density (g cm^−3^), and Ninorganic is the soil inorganic N content, which is the sum of NO_3_^−^-N and NH_4_^+^-N contents (mg kg^−1^). Soil bulk density was 1.24 g cm^−3^ at 0–20 cm, 1.31 g cm^−3^ at 20–40 cm, 1.41 g cm^−3^ at 40–60 cm, 1.40 g cm^−3^ at 60–80 cm, and 1.38 g cm^−3^ at 80–100 cm.


(7) $$Apparent\;N_{mine}=N_{uptake}+N_{residual}-N_{initial}$$

where apparent N mineralization (*N_mine_*, kg ha^−1^) is estimated based on the CK; *N_uptake_* is the N uptake in aboveground dry matter (kg ha^−1^); *N_residual_* and *N_initial_* are soil inorganic N accumulation after harvest and before sowing, respectively, in the 0 to 100 cm soil layer.


(8) $$Apparent\;N_{loss}=N_{mine}+N_{fertilizer}+N_{initial}-N_{uptake}-N_{residual}$$

Where *N_mine_* is calculated from the CK treatment; and *N_uptake_*, *N_residual_*, and *N_initial_* are calculated from a fertilization treatment.

Significant differences (*P*< 0.05) among N application treatments were determined using Tukey’s multiple range tests in SPSS 19.0 software (SPSS Inc., Chicago, IL, USA).

## 3 Results

### 3.1 Maize yield and yield components

Split-N fertilizer management significantly affected maize yield, kernel number per ear, and 1000-kernel weight (**Table 2**). Split-N application (N2, N3, N4, and N5) significantly increased maize yield (*P*< 0.001) compared with that with single basal fertilization (N1), with average increases of 13.8 to 24.5% over the three years. In 2018, increases ranged from 13.3 to 25.1%; in 2019, they ranged from 11.9 to 21.9%; and in 2020, they ranged from 16.2 to 26.6%. Differences in yield were due to significant increases in kernel number per ear (*P*< 0.001) and 1000-kernel weight (*P*< 0.001) in split-N treatments, with average increases ranging from 6.4% to 13.7% and 4.6% to 9.5%, respectively, over the three years.

**Table 2:** Maize yield and yield components in different nitrogen (N) application treatments.

Year	Treatment^a^	Kernel numberper ear^b^	1000-kernelweight (g)	Yield(kg ha^−1^)	Rate of growth (%)^c^
2018	CK	427.7 e	281.2 e	8029 e	
	N1	460.1 d	296.4 d	9450 d	17.7
	N2	482.1 c	315.7 c	10708 c	34.1
	N3	509.5 ab	324.2 b	11315 b	40.9
	N4	521.0 a	331.0 a	11824 a	47.3
	N5	504.2 b	323.4 b	11255 b	40.2
2019	CK	397.1 e	266.7 d	7072 e	
	N1	490.1 d	300.8 c	10173 d	43.8
	N2	514.9 c	312.4 b	11379 c	60.9
	N3	541.0 b	321.7 a	11937 b	68.8
	N4	550.9 a	327.3 a	12403 a	75.4
	N5	537.4 b	322.3 a	11720 b	65.7
2020	CK	339.6 d	256.3 e	5349 e	
	N1	456.8 c	308.8 d	9654 d	80.5
	N2	500.6 b	319.4 c	11218 c	109.7
	N3	520.5 ab	327.4 b	11825 ab	121.1
	N4	528.0 a	333.4 a	12221 a	128.5
	N5	518.7 ab	326.1 b	11671 b	118.2

a The total amount of N fertilizer applied in different proportions at sowing, sixth-leaf (V6), twelfth-leaf (V12), tasseling (VT), and blister (R2) stages of maize growth: N1, 100-0-0-0-0; N2, 50-50-0-0-0; N3, 30-50-20-0-0; N4, 20-30-30-20-0; and N5, 10-20-30-20-20. CK: no N fertilizer.

b Different letters within a year indicate significant differences among treatments (P < 0.05).

c Rate of growth in N application treatments in each year was based on comparison with CK without N application.

Among split-N treatments, maize yield, kernel number per ear, and 1000-kernel weight first increased and then decreased with the increase in N application frequency. The highest yield was in N4 treatment, and compared with that in N2, N3, and N5 treatments, average maize yield increased by 11.8%, 3.9%, and 5.1%; kernel number per ear increased by 6.8%, 1.8%, and 2.5%; and 1000-kernel weight increased by 4.6%, 1.8%, and 2.1%, respectively, over the three years.

### 3.2 Nitrogen uptake

**Figure 2:**
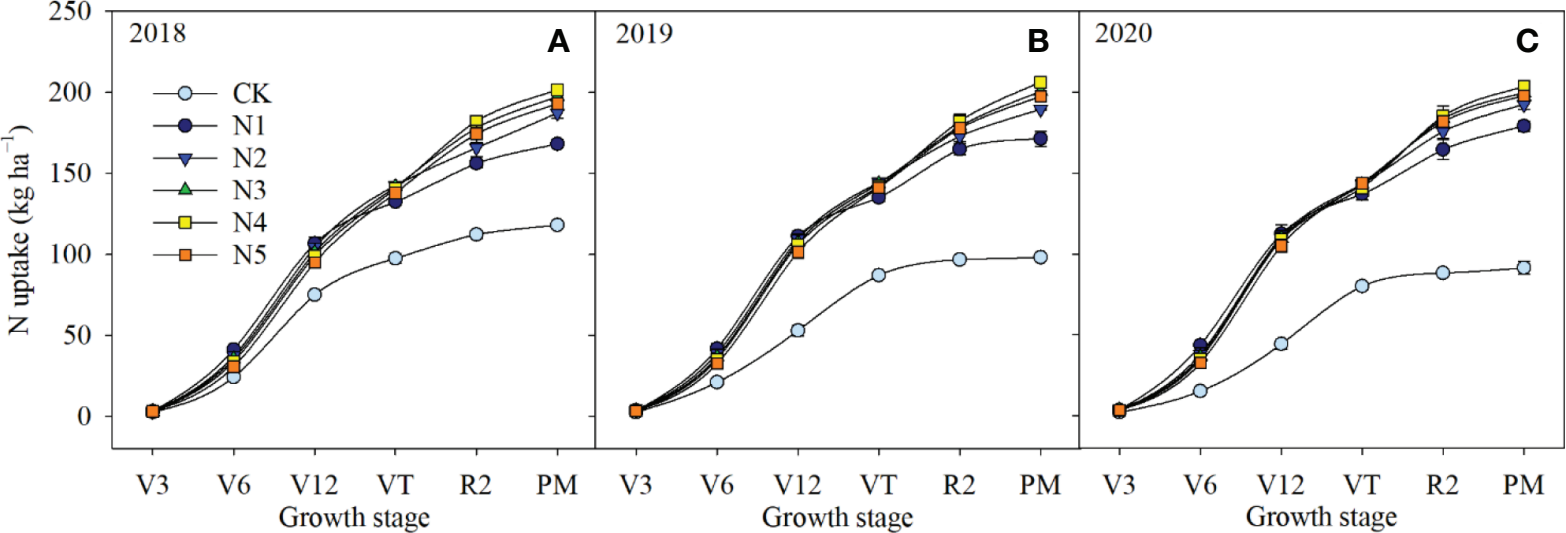
Nitrogen (N) uptake in maize at different growth stages in different N application treatments in 2018 **(A)**, 2019 **(B)**, and 2020 **(C)**. Growth stages: V3, third-leaf; V6, sixth-leaf; V12, twelfth-leaf; VT, tasseling; R2, blister; PM, physiological maturity. Error bars represent the standard deviation.

Nitrogen fertilizer applications increased N accumulation from V6 to PM stages compared with that in CK, although there were no differences in the V3 stage (**Figure 2**). Among N application treatments, there were no differences in N accumulation at the V3 stage, but N accumulation in N1 was higher than that in the split-N treatments (N2, N3, N4, N5) from V6 to V12 stages. However, from VT to PM stages, N accumulation was lower in N1 than in split-N treatments. The highest N accumulation from V12 to VT stages was in N2, which indicated that topdressing at the V12 stage promoted an increase in N accumulation. By contrast, the highest N accumulation from VT to PM stages was in N4, indicating that applying part of the N fertilizer to V6, V12, and VT stages could maintain a higher rate of N accumulation in late stages. However, topdressing in the R2 stage did not significantly affect N accumulation. At maturity, aboveground N accumulation in N4 increased by 7.4%, 2.3%, and 3.9% compared with that in N2, N3, and N5, respectively.

**Figure 3:**
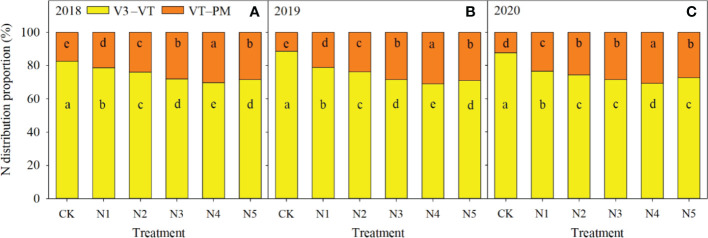
Nitrogen (N) distribution in different growth periods in different N application treatments in 2018 **(A)**, 2019 **(B)**, and 2020 **(C)**. Different letters in columns for each growth period in each year indicate significant differences in the proportion of N (*P* < 0.05). V3, third-leaf; VT, tasseling; PM, physiological maturity.

Nitrogen fertilizer significantly increased the proportion of total N accumulation in VT to PM stages compared with that in CK (**Figure 3**). In addition, the proportion of total N accumulation in VT to PM stages was 11.2% to 39.6% higher in split-N treatments (N2, N3, N4, and N5) than in the single basal fertilization treatment (N1) over the three years. However, the proportion of total N accumulation in VT to PM stages first increased and then decreased with increasing frequency of N application. The highest proportion was 30.7% in N4, increasing by 25.6%, 8.4%, and 8.5% compared with that in N2, N3, and N5, respectively.

**Figure 4:**
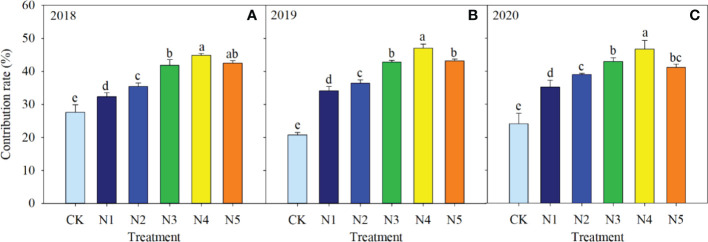
Contribution (%) of accumulated nitrogen (N) after the tasseling stage to grain N in different N application treatments in 2018 **(A)**, 2019 **(B)**, and 2020 **(C)**. Different letters above columns in each year indicate significant differences in contribution (*P* < 0.05).

Split-N treatments significantly increased the contribution of accumulated N to grain N accumulation after the VT stage, with average increases of 9.2% to 36.6% compared with the N1 treatment over the three years (**Figure 4**). Among different split-N treatments, contribution of accumulated N after the VT stage to grain N first increased and then decreased with the increase in N application frequency. The highest contribution was in N4, which increased by an average of 25.1%, 8.6%, and 9.3% compared with that in N2, N3, and N5, respectively, over the three years.

### 3.3 Nitrogen use efficiency

**Figure 5:**
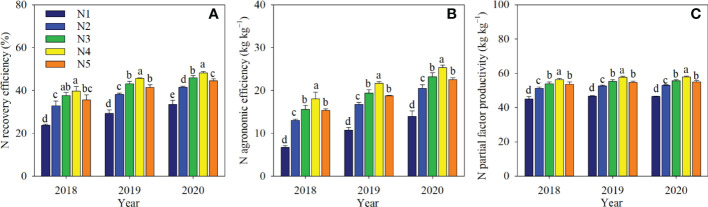
N recovery efficiency **(A)**, N agronomic efficiency **(B)**, and N partial factor productivity **(C)** in different N application treatments in 2018, 2019, and 2020. Different letters above columns in each year indicate significant differences in N efficiency (P< 0.05). Error bars represent the standard deviation.

Accumulated recovery efficiency (REN), AEN, and PFPN were significantly affected by split-N applications (**Figure 5**). Compared with N1, split-N treatments significantly increased REN by 8.0–14.7 percentage points, AEN by 6.5–11.4 kg kg^−1^, and PFPN by 6.5–11.4 kg kg^−1^ over the three years. Among split-N treatments, the highest N use efficiency was in N4, and compared with N2, N3, and N5, REN increased significantly by 6.7, 2.2, and 3.6 percentage points; AEN by 4.9, 2.2, and 2.9 kg kg^−1^; and PFPN by 4.9, 2.2, and 2.9 kg kg^−1^, respectively.

### 3.4 Regression analysis between N accumulation and yield and N use efficiency

**Figure 6:**
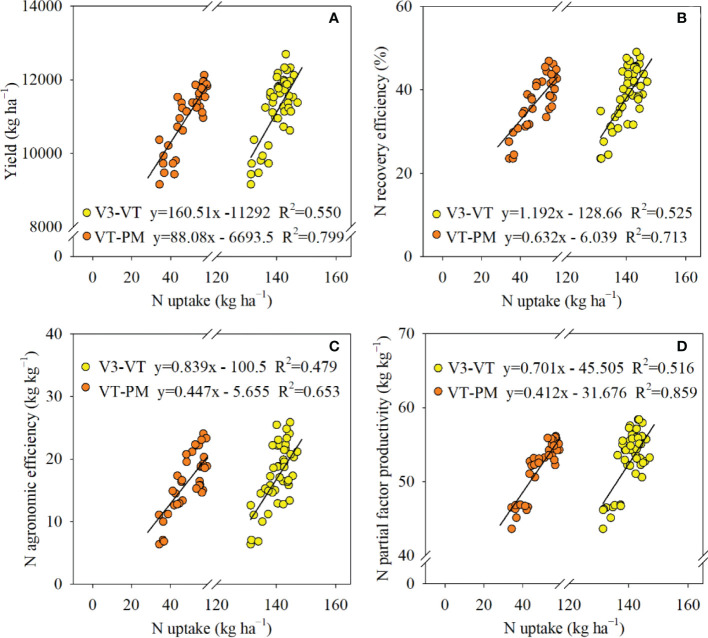
Linear regressions between N uptake and yield **(A)**, N recovery efficiency **(B)**, N agronomic efficiency **(C)**, and N partial factor productivity **(D)** before (V3–VT) and after (VT–PM) tasseling stages of maize in 2018, 2019, and 2020. Growth stages: V3, third-leaf; VT, tasseling; PM, physiological maturity.

Regression analyses showed that yield, REN, AEN, and PFPN were significantly positively related to N accumulation in V3 to VT and VT to PM stages (**Figure 6**). However, correlation coefficients of linear equations for VT to PM stages [*R* ^2^ = 0.799 (yield), 0.713 (REN), 0.653 (AEN), 0.859 (PFPN)] were higher than those of equations for V3 to VT stages [*R* ^2^ = 0.550 (yield), 0.525 (REN), 0.479 (AEN), 0.516 (PFPN)], which indicated that N accumulation was more closely related to yield and N use efficiency in VT to PM stages.

### 3.5 Soil inorganic N content and apparent N balance

**Figure 7:**
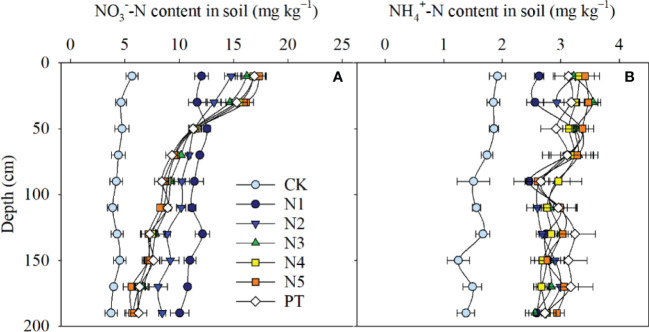
NO_3_^−^-N content **(A)** and NH_4_^+^-N content **(B)** at 20 cm intervals from 0 to 200 cm after maize harvest in different N application treatments over three years. PT, contents at beginning of experiment. Error bars represent the standard deviation.

Split-N treatments (N2, N3, N4, N5) significantly increased soil NO_3_^−^-N content by 22.9% to 57.1% at 0–20 cm and by 19.8% to 52.7% at 20–40 cm compared with N1 (**Figure 7**). In the two surface layers, NO_3_^−^-N content tended to increase with the increase in N application frequency, with the highest NO_3_^−^-N content in N5. However, the opposite trend was observed in 40–200 cm soil layers, with the highest NO_3_^−^-N content in N1. Among split-N treatments, the highest NO_3_^−^-N content at 40–200 cm was in N2, whereas there were no significant differences among N3, N4, and N5. Compared with contents at the beginning of the experiment, N1 and N2 treatments reduced NO_3_^−^-N content in the 0–40 cm soil layer but increased it in the 60–200 cm layer, whereas there were no differences in N3, N4, and N5 treatments.

Soil NH_4_^+^-N content in split-N treatments (N2, N3, N4, and N5) was significantly higher than that in the N1 treatment at 0–40 cm (*P* = 0.002–0.037), but no differences were detected at 40–200 cm (*P* = 0.054–0.723). Compared with contents at the beginning of the experiment, NH_4_^+^-N content was not significantly different in different soil layers in any treatment (*P* = 0.064–0.726), except in N1, which had low NH_4_^+^-N content in the 0–40 cm soil layer.

The apparent N balance in the soil–plant systems is shown in **Table 3**. Split-N application increased crop N uptake and soil inorganic N sequestration by 9.8% to 17.9% and 2.5% to 6.6%, respectively, compared with the N1 treatment. The highest N uptake was in N4 treatment, whereas the highest soil accumulation at harvest was in the N5 treatment in 2020. Compared with the N1 treatment, split-N treatments significantly reduced the apparent N loss by 18.1% to 33.7%, with the largest reduction in N4. In the N4 treatment, apparent N loss was reduced by 19.1%, 6.8%, and 9.3% compared with that in N2, N3, and N5 treatments, respectively. Thus, split-N applications significantly improved N uptake and soil inorganic N accumulation at harvest and substantially reduced N losses when the N fertilizer application rate was optimized in major periods of maize growth.

**Table 3:** Apparent nitrogen (N) budget for the 0 to 100 cm depth in the plant–soil system in different N treatments over three years (2018 to 2020).

Parameter	N1	N2	N3	N4	N5
Soil N accumulation before experiment (kg ha^−1^)	206.9	206.9	206.9	206.9	206.9
Apparent N mineralization (kg ha^−1^)	188.2	188.2	188.2	188.2	188.2
Fertilizer N input (kg ha^−1^)	630	630	630	630	630
Crop N removal (kg ha^−1^)	518.6 d	569.3 c	597.5 b	611.2 a	588.3 b
Soil N accumulation after experiment (kg ha^−1^)	198.6 c	203.6 bc	208.7 ab	209.8 ab	211.7 a
Apparent N loss (kg ha^−1^)	307.9 a	252.2 b	218.9 c	204.1 d	225.1 c

Different letters in the same row indicate significant differences among N application treatments (P < 0.05).

The system was film-mulched drip-irrigated maize in Northeast China.

## 4 Discussion

### 4.1 Effects of different drip irrigation N application strategies on maize yield and yield components

Exogenous N supply significantly affects crop yield and its components (Fois et al., 2009; Rolbiecki et al., 2021), and regulating N fertilizer delivery can further increase those effects (Overman and Scholtz, 1999). In maize, the effective number of panicles is unaffected by the N application method (Zheng et al., 2016), and thus, maize yield is determined by kernel number per ear and 1000-kernel weight. A single basal N application can lead to early seedling burn and collapse, as well as insufficient fertilizer supply in later growth stages, which ultimately reduce maize yield (Gao et al., 2007; Walsh et al., 2012). However, partial amounts of total N fertilizer applied in middle and late growth stages can increase kernel number per ear and 1000-kernel weight, and thus, maize yield increases with the same amount of N fertilizer (Fan et al., 2020). Although split-N application can solve the problems with a single basal N application, topdressing doses in different growth stages need to be determined. In the current study, when N fertilizer application was concentrated in V6 and V12 stages (N2 and N3 treatments); yield increased compared with a single basal application (N1 treatment). However, the N supply was insufficient in later stages, which can reduce maize yield (Wang et al., 2019). By postponing 20% of the total N fertilizer to the VT stage, the N4 treatment compensated for N deficiency from the flowering to maturity stage, which had a positive effect on promoting maize filament growth, improving the grain filling rate, increasing kernel number per ear and 1000-kernel weight, and ultimately obtaining high yields. However, a higher ratio of postponing N application was not better. For example, in this study, the N5 treatment postponed 40% of the total N fertilizer to the VT and R2 stages. Under the low N fertilizer supply in the early stage, nutritional growth development was inhibited to some extent, and panicle differentiation quality was affected. Therefore, the yield in N5 was lower than that of N4 treatment. Therefore, to increase kernel number per ear and 1000-kernel weight and ultimately achieve high maize yields (Fan et al., 2005), N fertilizer application must meet N demand in the early stage of growth while ensuring the supply of soil N is sufficient in the late stage of growth (Liu et al., 2018).

### 4.2 Effects of different drip irrigation N application strategies on maize N uptake and utilization

Crop growth, root absorption capacity, and changes in soil N availability affect crop accumulation of N (Soufizadeh et al., 2018). Under optimal fertilizer application, the increase in N accumulation directly contributes to increases in crop yield and N fertilizer use efficiency (Zheng et al., 2016). In maize, N uptake rate is low in the early growth stage before stage V6 when growth is not limited by water and nutrients (Guo et al., 2016). Thereafter, the rate of root uptake increases rapidly with increasing root biomass, with the peak at approximately the VT stage. Nitrogen requirements then decline from VT to PM stages (Overman and Scholtz, 1999).

In maize production, grain yield is closely related to N accumulation in the late growth stage (Yan et al., 2015). Thus, to improve crop yield and N use efficiency, fertilizer N supply should be synchronized with maize N requirements in that stage (Tian et al., 2018). In the current study, coefficients of determination of linear regressions between N accumulation and grain yield and N use efficiency were higher from VT to PM stages than from V3 to VT stages. Those results further demonstrated that increasing N accumulation after the VT stage increased maize yield and N fertilizer use efficiency more than increasing N accumulation before the VT stage. Split-N application significantly increased N accumulation from VT to PM stages compared with that with a single basal application, whereas some differences were observed between split-N treatments. In the current study, total N accumulation decreased in N2 and N3 treatments, because when fertilizer application was concentrated in V6 and V12 stages, N fertilizer supply decreased in the R2 stage. However, the N5 treatment, with the highest frequency of fertilizer application and the largest proportions applied in late stages, was also detrimental to N accumulation, because low doses of N fertilizer in the early stages inhibited N uptake for vegetative growth. In contrast, in N4 treatment, synchronization improved between N application strategy and maize N demand, because N demand was met in the early growth stage and higher plant N content was ensured after the VT stage. Split-N application with optimal rates and timing can ultimately achieve both high yield and N use efficiency in maize by maintaining stable leaf chlorophyll content, slowing down senescence of functional green leaf area, and increasing N accumulation after tasseling (Ding et al., 2011).

### 4.3 Effects of different drip irrigation N application strategies on N losses

Soil inorganic N (NO_3_^−^-N and NH_4_^+^-N) is the main form of N uptake by plants and is also easily leached from soil in one of the main pathways of N loss in rain fed maize cropping systems (Liu et al., 2003). Reducing N losses is one of the necessary conditions to improve N fertilizer use efficiency (Zheng et al., 2016). Soil N deficits and excesses are common and are caused by mismatches in N demand and soil N supply at different stages of growth (Shi et al., 2012). Reducing retention time in soil before crop uptake can reduce the risk of N loss and increase N use efficiency (Malhi et al., 2001). Therefore, adjusting times of N fertilizer application can effectively improve synchronization between soil N supply and crop absorption (Shi et al., 2012). In the current study, soil inorganic N contents in the split-N treatments were higher than that in the single basal application treatment at 0–40 cm, but the opposite was observed at 60–200 cm. Thus, the N supply with a single basal application not only far exceeded crop demand but also exceeded soil sequestration capacity at the early stage, which greatly increases the risk of N leaching (Guo et al., 2021). By contrast, split-N management increased the effective supply of soil N by increasing soil inorganic N content in the tillage layer at different growth stages, which increased crop uptake while decreasing deep soil inorganic N content and possibly reducing N loss. However, there were differences in N losses among split-N treatments. The apparent N loss with optimal application times and doses (N4) was significantly lower than that in other treatments (7.2% to 19.1%), whereas N uptake increased by 2.5% to 7.4%. This is because the N2 and N3 treatments concentrated the N supply at the V6 and V12 stages, resulting in an N surplus in the early growth stage. The unabsorbed N easily leaches into the deeper soil layers through gravitational percolation. However, the N5 treatment increased N losses by supplying a disproportionately high proportion of N in the late growth stage, which far exceeded the N demand of maize at that stage. N4 treatment reduced N loss by synchronizing maize N demand with soil N supply. In conclusion, a reasonable N fertilizer application strategy can not only promote N uptake but also effectively reduce N fertilizer losses and negative effects on the environment (Kühling et al., 2021).

## 5 Conclusions

In the semiarid region of Northeast China, a drip irrigation system combined with split-N fertilizer application increased maize yield by increasing kernel number per ear and 1000-kernel weight. It also improved N uptake in the later stage of growth and N use efficiency by matching soil N supply with maize N requirements. Split-N application reduced the risk of soil N leaching by increasing inorganic N content in the tillage layer and decreasing inorganic N content in deep soil. Among split-N treatments, the drip irrigation N application strategy with 20% basal fertilizer + 30% jointing fertilizer + 30% belling fertilizer + 20% tasseling fertilizer obtained the highest maize yield and N use efficiency and also reduced potential N losses. Thus, the N4 treatment has broad application prospects.

## Data availability statement

The original contributions presented in the study are included in the article. Further inquiries can be directed to the corresponding authors.

## Author contributions

YH conceived the study and wrote the manuscript. LK and LZ measured experimental samples. YZ analyzed data. XX and ZL designed the study and drafted the manuscript. All authors reviewed the manuscript. All authors contributed to the article and approved the submitted version.
